# Emergency medicine clerkship curriculum in a high-income developing country: methods for development and application

**DOI:** 10.1186/s12245-018-0190-y

**Published:** 2018-06-07

**Authors:** Arif Alper Cevik, Elif Dilek Cakal, Fikri M. Abu-Zidan

**Affiliations:** 1 Department of Internal Medicine, Emergency Medicine Clerkship, College of Medicine and Health Sciences, United Arab Emirates University, Al Ain, 17666 United Arab Emirates; 2 Department of Emergency Medicine, Tawam-John Hopkins Hospital, Al Ain, United Arab Emirates; 3 Department of Emergency Medicine, Mersin State Hospital, Mersin, Turkey; 4 Department of Surgery, College of Medicine and Health Sciences, United Arab Emirates University, Al Ain, United Arab Emirates

**Keywords:** Emergency medicine, Undergraduate, Curriculum

## Abstract

**Background:**

The published recommendations for international emergency medicine curricula cover the content, but exclude teaching and learning methods, assessment, and evaluation. We aim to provide an overview on available emergency medicine clerkship curricula and report the development and application experience of our own curriculum.

**Methods:**

Our curriculum is an outcome-based education, enriched by e-learning and various up-to-date pedagogic principles.

**Results:**

Teaching and learning methods, assessment, and evaluation are described. The theory behind our practice in the light of recent literature is discussed aiming to help other colleagues from developing countries to have a clear map for developing and tailoring their own curricula depending on their needs. The details of our emergency medicine clerkship will serve as an example for developing and developed countries having immature undergraduate emergency medicine clerkship curricula. However, these recommendations will differ in various settings depending on available resources.

**Conclusions:**

The main concept of curriculum development is to create a curriculum having learning outcomes and content relevant to the local context, and then align the teaching and learning activities, assessments, and evaluations to be in harmony. This may assure favorable educational outcome even in resource limited settings.

**Electronic supplementary material:**

The online version of this article (10.1186/s12245-018-0190-y) contains supplementary material, which is available to authorized users.

## Background

Each medical student must be competent in basic acute care medicine [1, 2]. Emergency medicine clerkships provide unique experiences to improve students’ confidence in managing acute care situations [3]. Although providing and teaching acute care is as old as medicine itself, the earliest formal undergraduate curriculum recommendations by Society of Teachers of Emergency Medicine stating the minimum standards in undergraduate emergency medicine (EM) education were made in the 1980s [4]. Since then, various organizations from developed countries have published undergraduate EM curricula [5–8].

The conception of curricula, evolved from a syllabus-like, content-based perspective to ones defining various components including purposes, experiences, methods, and evaluation [9, 10]. The aforementioned organizations have limited their recommendations covering the content of a curriculum, but excluded teaching and learning (TL) methods, assessment, and evaluation. The efforts to specify the minimum standards of undergraduate EM curriculums are short in these aspects.

The development process of our EM clerkship started at June 2013 and the current form of the curriculum was developed at the end of the academic year of 2016–2017. It was a 4-year continuous development process. We acknowledge that the above published recommendations discuss deeply the outcomes and the content of a standardized undergraduate EM curriculum. This article provides details of an emergency medicine clerkship as an example for developing and developed countries having immature undergraduate emergency medicine clerkship curriculum. In this communication, we aim to share our example of EM clerkship curriculum including TL methods, assessment, and evaluation, and finally discuss the theoretical knowledge behind our practice so as to help other colleagues in other countries to have a clear map for developing their own curricula.

## Methods

### UAEU undergraduate emergency medicine curriculum

United Arab Emirates University (UAEU), College of Medicine and Health Sciences (CMHS) EM clerkship for the final (sixth) year medical students is a 4-week rotation. There are five groups of medical students in the final year and each group consists of 13–18 students. EM clerkship is their first exposure to acute care medicine taught as a complete block.

The implementation of this curriculum requires personnel and facilities. Several groups of professionals share the learning and teaching responsibilities as tutors including one clerkship director, four core-faculty members of the EM residency program, and seven fourth-year EM residents. Most of the educational sessions take place in a simulation center and skills laboratory. Classroom includes smart board technology, computer, white board, and TV. There are two high-fidelity mannequins, and multiple types of low-fidelity mannequins and basic medical models for skill practices. Two hospitals (Al Ain and Tawam Hospitals) provide the clinical environment.

**Fig. 1:**
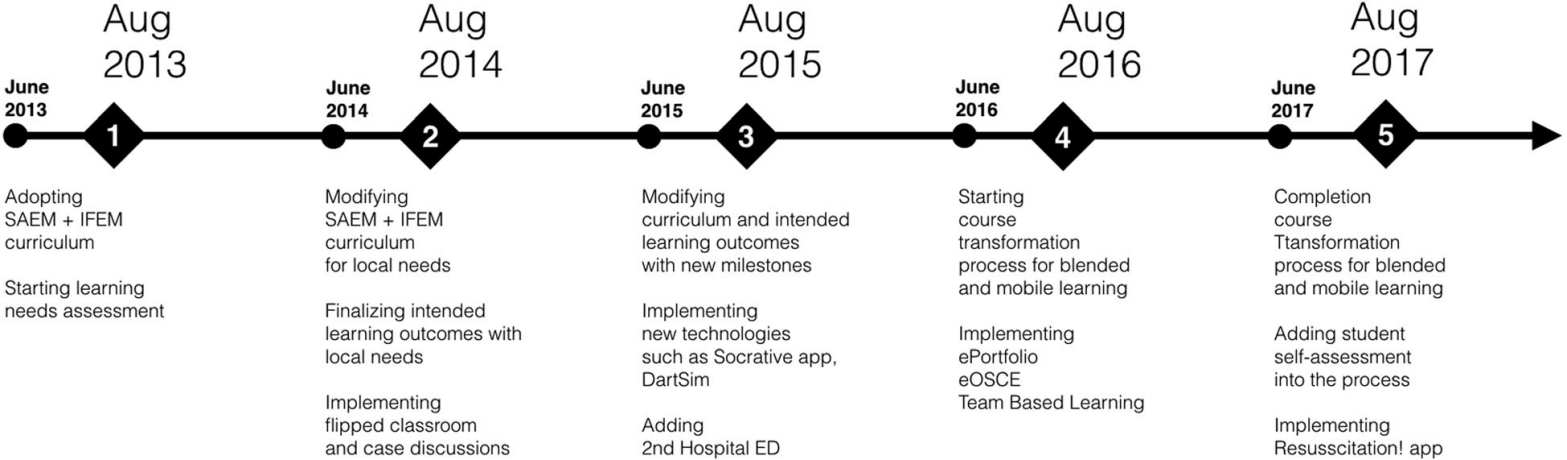
Emergency medicine clerkship curriculum development time table. Undergraduate EM curriculum development started in June 2013. The curriculum modification was applied in every June, and the updated curriculum was used at the beginning of each new academic year (in August). Numbers in the black rectangles represent the year of UAEU-undergraduate EM curriculum development. Black circles represent curriculum modification months just after the academic year has finished. All modifications were completed during the summer time when there were no senior student groups

Undergraduate EM curriculum is fundamentally  outcome-based, enriched by e-learning and various pedagogic principles. Its development started in June 2013, and the first group of student was enrolled in September 2013. Figure 1 shows the milestones in the process. Table 1 shows general structure of the 4 weeks EM clerkship.

**Table 1:** General 4-week schedule of the EM clerkship

Week -1	D -7				D -3		D -1
	Emergency medicine clerkship curriculum is sent to students				Entry self-assessment survey is conducted		Students’ subgroups^a^ are defined, and OneNote invitation is sent to students
Week 1	D1	D2	D3	D4	D5	D6	D7
	08:00–16:00 Introduction to emergency medicine clerkship TBL activities and case discussions	Clinical shifts	Clinical shifts Subgroup A cannot have night shift	08:00–12:00 Emergency medicine residency program teaching day for subgroup A Clinical shifts for other subgroups (except night time)	08:00–12:00 Simulation and skills practice sessions Weekly group feedback session	Clinical shifts	Clinical shifts (except night time)
Week 2	D8	D9	D10	D11	D12	D13	D14
	08:00–16:00 TBL activities and case discussions	08:00–12:00 Simulation and skills practice sessions Weekly group feedback session	Clinical shifts Subgroup B cannot have night shift	08:00–12:00 Emergency medicine residency program teaching day for subgroup B Clinical shifts for other subgroups (except night time)	08:00–13:00 Individual feedback session	Clinical shifts	Clinical shifts (except night time)
Week 3	D15	D16	D17	D18	D19	D20	D21
	08:00–16:00 TBL activities and case discussions	Clinical shifts	Clinical shifts Subgroup C cannot have night shift	08:00–12:00 Emergency medicine residency program teaching day for subgroup C Clinical shifts for other subgroups (except night time)	08:00–12:00 Simulation and skills practice sessions Weekly group feedback session	Clinical shifts	Clinical shifts (except night time)
Week 4	D22	D23	D24	D25	D26	D27	D28
	08:00–16:00 TBL activities and case discussions Simulation and skills practice sessions Exit self-assessment survey is conveyed	Revision session Information regarding MCQ and OSCE Free skills practice session Clinical shifts (except night time)	Clinical shifts (except night time)	13:00–15:30 Final MCQ exam	08:00–12:00 OSCE Final feedback Course evaluation survey	Clinical shifts (if necessary)	Clinical shifts (if necessary)

*D* day, *MCQ* multiple choice question, *OSCE* objective structured clinical examination, *TBL* team-based learning

^a^Subgroups consist 4–6 students

## Results

### Intended learning outcomes and content

The intended learning outcomes (ILO) and content were gathered from two resources. First, the previously published frameworks by Society for Academic Emergency Medicine and International Federation for Emergency Medicine curriculums provided the core structure [6, 7]. The UAEU-undergraduate EM curriculum was further revised in 2014 based on EM student milestones [11]. Second, learning needs assessment was applied to detect local needs. The feedback from stakeholders, including medical students, faculty members, EM residents, nurses, and patients were collected via surveys. These included either paper-based surveys, electronic based surveys, or face-to-face interviews which were collected during the 2013–2014 academic year. Feedbacks have been continuously collected via electronic surveys and face to face interviews for quality improvement. The main results of feedbacks and proposed improvements for the curriculum are shown in Additional file 1: Appendix 1. The final version of UAEU-undergraduate EM curriculum includes eleven chief complaint and six procedure topics (Additional file 1: Appendix 2).

### Teaching and learning methods

There are five teaching and learning (TL) aspects of our curriculum; classroom activities, skills and simulation laboratory sessions, clinical exposure, feedback, and the online platform. The use of e-learning tools is a substantial part of our TL activities. However, it does not decrease face-to-face time between faculty/tutors and students. Therefore, this format is a technology-enhanced learning [12].

#### Classroom activities

Classroom activities are divided into two broad groups according to their topics: (a) chief complaint-related and (b) procedural. Case discussions, flipped classroom, and team-based learning (TBL) form the basis for the chief complaint-related activities. The aim of the chief complaint-related activities is mainly to facilitate students’ differential diagnosis on the concept of ruling out the worst-case scenario, critical decision-making processes, and case management. Factual knowledge is not a core objective during chief complaint-related sessions. The students are expected to attend these sessions prepared by studying recommended pre-class materials regarding each chief complaint. This includes instructor-made videos, university’s online library resources, and external web links (video, web page, and online textbooks). Eight out of 11 chief complaint topics are delivered via TBL sessions. Each TBL session is 90 min. The rest three topics are provided as case discussions. The duration of each case discussion session is 60 min. This includes three to four real-life case scenarios. Case discussions are facilitated by clerkship director, core-faculty members, or senior (fourth year) EM residents. Case discussions, flipped classroom, and TBL have been, respectively, implemented during 2013–2014, 2015–2016, and 2016–2017 academic years.

TBL is a variation of flipped classroom [13, 14]. It includes three stages [15, 16]. In stage 1, students prepare individually for the class. In stage 2, they take an individual readiness assurance test (i-RAT). Immediately after the i-RAT, permanent groups of six to seven students take the same test. This is called group or team readiness assurance test (t-RAT). Group members discuss the questions to reach the best answers. Both i-RAT and t-RAT provide information about learning gaps. In stage 3, detected learning gaps are addressed via collaborative group activities called application exercise. In our curriculum, case scenarios are the main method used in this stage.

Lectures presented in the classroom are only related to procedural topics. This includes Extended Focused Assessment with Sonography in Trauma (E-FAST) and Rapid Ultrasonography in Shock and Hypotension (RUSH) protocol. These lectures are given by the clerkship director. The practical sessions of these procedures are held separately.

#### Skills and simulation laboratory sessions

There are six procedures covered in the curriculum: cardiac arrest and arrhythmia management, airway management, suturing, intraosseous line insertion, E-FAST, and RUSH protocols. Students are expected to prepare for the sessions. We are using classroom teaching only for EFAST and RUSH protocols. Each practical session (100 min) consists an introductory presentation for 10 min followed by supervised practice on high-fidelity manikins, skills training models or simulated patients for 90 min. Additional supervised and non-supervised practice sessions are available as needed (Additional file 1: Appendix 2). We used Gagne et al.’s instructional design for procedure education [17]. These levels are the following: level 1, gaining attention; level 2, informing learner of objectives; level 3, stimulate recall of prior learning; level 4, presenting stimulus; level 5, providing learning guidance; level 6, eliciting performance; level 7, providing feedback; level 8, assessing performance; and level 9, enhancing retention and transfer.

#### Clinical exposure

Students have to complete 10 clinical shifts. The 9-h shifts are different depending on the location, ED section, ED unit, time, supervision, clinical severity, and learning experience. Students rotate in two main hospitals, each treating over 115,000 emergency patients annually. One of these hospitals provides EM residency program. Students are assigned to adult and pediatric ED sections. They undertake shifts in resuscitation, urgent care, and fast-track units. Students participate in different time of shifts including day time (08:00–17:00), prime time (13:00–22:00), evening time (15:00–24:00), and night time (23:00–08:00) under the supervision of senior EM residents and attending emergency physicians. These include core faculty members. Ideally, a maximum of two students are allowed to be in one unit per shift to ensure enough patient and procedure exposure for each student. Occasionally, this rule was not followed because of the relatively large number of students.

#### Feedback

Feedback is at the core of UAEU-undergraduate EM curriculum. Several types of feedback from various resources are available during this clerkship. First, self-assessment surveys provide an opportunity for self-reflection. Two surveys, one at the beginning and one at the end of the TL activities, are conducted to induce insights into the students’ strength and weakness regarding ILOs. Additionally, the TL activities are slightly modified to address personal anticipated needs according to the survey results.

Second, brief feedback is an integral part of all classroom and skills practice sessions. These sessions are designed to enable peer and tutor feedback. Tutors provide feedback during the case discussions and skill sessions. Under-achieved areas are detected during discussions and reviewed at the end of the sessions. Furthermore, the highlights of each session are shared on a collaborative online platform (OneNote).

Third, individual and group feedback sessions are held on alternate weeks. These two-way feedback sessions are facilitated by the clerkship director. The duration of each group session is 30 min. The duration of individual feedback session is 20 min per student. Both types of sessions focus on the review of students’ learning activities, clinical performance, TBL assessment results, and areas that need improvement as well as the students’ recommendations to improve the program.

Fourth, the collaborative online platform supports feedback in different ways. The summary of the feedback on the under-achieved areas is published online after each session. Furthermore, the clerkship director weekly creates additional group-specific tasks and case discussions to induce discussion on areas that need improvement. The clerkship director provides his online feedback on the students’ responses for the weekly cases so as to close the learning gap. This feedback is available for the whole group to enhance learning and promote self-reflection.

#### Online platforms and supportive applications

*Online platforms*

Undergraduate EM curriculum contains a number of online platforms to enhance learning experiences such as Google Forms, Socrative app, Microsoft OneNote, and SharePoint.

*Google forms*

Self-assessment surveys, evaluations of student case presentations, tutor case discussions, and faculty teaching performance are conducted via Google Forms [18]. Google Forms provides multiple advantages including easiness to create, share, and analyze with free of charge.

*Socrative*

Socrative [19] is mainly used for assessments during TBL sessions and during clerkship final evaluation surveys. Socrative is a free online platform. It is compatible with several mobile devices and provides several question types including true-false, multiple choice, and short answer questions. One of the advantages of this platform is the real-time observation of the answers.

*OneNote*

OneNote [20] provides a private virtual classroom environment. Tutors and invited students can establish a two-way or group communication. The details of the curriculum and ILOs, additional weekly case discussions, case discussion highlights, self-assessment surveys’ results, student case presentations, and students’ selected clinical cases can be shared on OneNote. This platform needs institutional membership to provide online classroom environment.

*SharePoint*

SharePoint [21] is the electronic logbook (e-Portfolio) medium of UAEU undergraduate EM curriculum. Students need to record every case and procedure they perform during clinical shifts. Additionally, this platform hosts students’ shift evaluation and supervisors’ student evaluation, which need to be completed after each shift. These results are continuously monitored by the clerkship director to feedback the students, supervisors, and staff.

##### Supportive applications

*DartSim*

DartSim [22] mimics the functions and audio-visual features of a real defibrillator and monitor, so it enables to imitate a more realistic ED environment. Displayed on the screen, there are vital signs, ECG rhythm strip, buttons for a 12-lead ECG, defibrillator, CPR, pacing, patient X-ray, and drugs options which can be manually managed by students or remotely by the tutor.

*Resuscitation!*

Resuscitation! [23] provides more than 100 case scenarios for EM-related chief complaints. Scenarios start with only a case vignette. Students lead the scenario to solve the case. The application provides vital signs monitoring, examination, diagnosis, order choices grouped from critical to harmful options. The choices made are recorded by the application for review.

*eOSCE*

The clerkship director uses Electronic Objective Structured Clinical Evaluation (e-OSCE) desktop program to compose evaluation sheets for OSCE exams [24]. Examiners may evaluate and record the students’ performance using these sheets via e-OSCE application on iOS tablets. The clerkship director can remotely monitor the results on real-time. Once the evaluation forms are downloaded from the cloud, this validated application does not need a continuous internet connection to work [24]. All information collected can be uploaded to the cloud when the Wi-Fi connection is re-established.

### Assessments

UAEU undergraduate EM curriculum contains multiple formative and summative assessments. The formative assessments mainly include the answers to the additional weekly cases in OneNote. However, flipped classroom with case discussions, TBL sessions, and skills practice sessions were used as formative feedback tools. There are six summative assessments in the clerkship. Forty percent of summative assessments are from OSCE and final MCQ exam. The details of summative assessments are summarized in Additional file 1: Appendix 3.

### Evaluation of the program

Individual and group feedback sessions, clerkship evaluation survey, senior residents’ and core faculty members’ feedback, and summative assessment results are taken into consideration when evaluating the program. The evaluation process is completed between June and July. Required changes are implemented to curriculum before the new academic year. All students’ groups are exposed to the same updated curriculum. There is no change applied during the academic year.

### Students’ exposure and need for revision sessions

Students start the clerkship with self-assessment entry survey regarding ILOs. They are exposed to 47 h of TL activities assisted by the clerkship director and tutors in the classroom and skills/simulation laboratory. During the classroom activities, we discuss 30 to 44 cases depending on the needs of the students. These cases are determined by the results of individual and team readiness assurance test of TBL activity. For example, if individuals or groups are lacking to reach an ILO by having wrong answers to questions, then this ILO is stressed during the case discussions of the application exercise step of TBL. We also share 16 additional cases in OneNote (Additional file 1: Appendix 4). The cases in the OneNote are shared to improve the understanding of the ILOs after the flipped classroom and TBL sessions. Groups also present their 13–18 case presentations. Students are free to choose their cases to be presented depending on their level and learning needs. This approach gives them an opportunity to master the knowledge of any topic they want. Students also share the cases they were exposed to during their clinical shifts. Their minimum case discussion exposure in the TL activities is 59 cases during the four weeks clerkship. Students are exposed to 120 MCQs during their TBL sessions before their final exam. Students experience practical skills for 13.5 h before their OSCE. They receive 90-min group feedback session and 20-min individual feedback session. Students’ clinical exposure is 90 h in which they see an average of 68.3 (SD 17.6) patients and perform an average of 46.1 (SD 14.0) procedures [25].

After the curriculum content is covered and all measures are tried to reach ILOs, students take an EM clerkship self-assessment exit survey which includes the same items they did as an entry survey. They re-assess their confidence levels regarding the ILOs. The results of the exit survey are reviewed by the clerkship director. The ILO items which received less than 80% achievement are listed on the revision day topics. Revision topics are discussed with the students one by one under the guidance of the ILOs. At the end of the revision session, students are provided with the information regarding the coming two exams, MCQ and OSCE. After this process, they are considered to be eligible to proceed for both exams.

## Discussion

We presented the UAEU undergraduate EM curriculum to serve as a model for clerkship directors in developing countries. Table 1, Additional file 1: Appendices 1–4 provide the details of schedule, stakeholders’ feedbacks, educational activities, assessments, and online feedbacks of tutors for case discussions, respectively.

Many valuable frameworks and resources regarding content and learning outcomes are available [7, 8, 11]. These frameworks are syllabus-like, excluding TL methods, assessment, and evaluation. Moreover, examples of a structured EM curricula are few in developing countries [26]. Although recommended topics and learning outcomes are applicable to different settings, a local learning needs assessment covering all stakeholders should be conducted to modify content according to context [27, 28].

Our outcome-based undergraduate EM curriculum is a blend of multiple educational trends and e-learning. In outcome-based education, course outcomes guide all curriculum plans and processes [29]. The content, its organization, educational environment, teaching and learning methods, assessments, and curriculum evaluation processes are defined under the guidance of the ILOs. Outcome-based education encourages shared responsibility for learning among the teachers and the students [29]. Teachers should assure that guidance, TL methods, and resources are sufficient to achieve the learning outcomes. In contrast, students are responsible for giving their effort to reach those outcomes.

Today, medical teachers are aware of the limited positive impact of large group lectures on deep learning [30]. Interactivity supports questioning and engagement during face-to-face sessions [30]. Interactive case discussions enhance learning by making connection between knowledge-based class activities and the real clinical environment [31]. Case discussions encourage self-regulated learning [32]. It also identifies the gaps in knowledge or skills [33] and gives the tutor a chance to provide feedback. Thus, in our curriculum, case discussions are in the center of flipped classroom and TBL sessions.

The flipped classroom is a blended approach in which learners prepare themselves for the topic independently in a learner-paced manner before the class with given videos and online resources [32, 34, 35]. Classroom time is dedicated to knowledge application, simulation, case-based learning [34]. This process also facilitates student-centered learning which allows the students to determine their specific learning goals [13]. Student-centered education encourages students explore the solutions without a complete dependence on a tutor or instructor learning and promotes life-long learning [36]. Although EM literature does not show a difference in gained knowledge [37], students and teachers prefer flipped classroom over traditional lecturing because of interactivity, discussions, active participation, and synthesized knowledge [35].

TBL is an interactive small group activity needing less resources which is directed by a teacher [38–40]. It has certain advantages over passive, didactic, lecture-based learning. It promotes critical thinking, problem-solving, team building and communication skills [41, 42]. Although many studies showed knowledge increases via TBL, Fatmi et al.’s systematic review highlighted the need for further studies regarding its effectiveness [43]. Unlike other established fields [43, 44], TBL in EM education is still developing [45, 46]. Our recent experience between 2015 and 2017 has shown that TBL improved knowledge-based performance both in clerkship and medical school exit exams with better long-term knowledge retention (unpublished submitted data, Cevik AA et al.).

E-learning creates a compelling learning environment which expands students’ thinking processes and knowledge [47]. It is flexible regarding pace, place, and mode, and it enables personalized learning [48]. It supports both synchronous and asynchronous learning [48]. e-Portfolio provides real-time data regarding students’ strengths and weaknesses at both individual- and group-level [48, 49]. It catalyzes data analysis. Another advantage of online learning environment in our curriculum is to provide continuous feedback to students. One of the significant advantages of using e-learning tools in our clerkship was guiding the students with continuous feedback regarding their e-Portfolio logs to achieve the curriculum recommendations [50]. Medical students highly valued the use of e-learning platforms even in the clinical skills education [51].

Technology is a tool to reach desired learning outcomes, and without adequate motivation, students may not completely engage with it [52]. Therefore, faculty members/tutors should generate interest and continuously monitor and support learning processes [52]. Some authorities state that technology makes learning more relevant, and thus, motivates students [53, 54]. Students express that technology enriches their learning experience, and they prefer blended learning over the face-to-face model [55]. Today, there is adequate evidence to show that students are motivated by technology-enhanced learning methods [52].

Yeung et al. reported that students appreciated receiving multiple feedback and evaluation from their tutors and supervisors during the EM clerkship [56]. Feedback is one of the major components of our curriculum. Without a goal or outcome-directed formative feedback, students’ achievements will be minimal [57]. Immediate feedback is powerful and efficient at correcting problems in knowledge and skills [58].

Assessment drives learning [59]. There are six different types of assessments in our curriculum (Additional file 1: Appendix 3). Although summative assessments have some value in feedback, formative assessment aims to provide useful feedback on student strengths and weaknesses concerning the ILOs [60]. Formative assessments motivate students, and motivation increases learning [61]. The main advantage of implementing multiple formative assessments is to measure student’s level of accomplishment and guide their learning by frequent feedback [62]. Clerkship directors should ensure that the ILOs, TL activities, and the assessment are aligned [63, 64].

## Limitations

We have to acknowledge that our curriculum has certain shortages. The process is time and resource consuming. Because of faculty shortage and rapid turnover rate of attending physicians and adjunct faculty members, application of the curriculum needs close clerkship director control. The extreme variations in attending physicians’ scoring and time constraints may hinder the sufficient number of assessments [65]. This has forced us to remove some proven validated bedside assessments such as Mini-Clinical Evaluation Exercise. Some authors claim that the required number of stations for reliable OSCEs is 16 [66]. However, the number of available examiners limits our number of stations to be only eight. On average, our students reach the required number of clinical cases and procedures. However, their distribution of exposure is still non-homogenous [25]. The high number of trainees and residents at different levels compete with our students on their learning opportunities. Accordingly, students have difficulty in reaching the planned targets even though they rotate in all available teaching hospitals in our city. This makes us think that four weeks may be a short period for medical students to grasp all needed skills to manage acute care medicine in our setting. In our format, i-RAT and t-RAT require the use of students’ mobile devices. This increases the risk of copying the questions. Therefore, a considerable part of the clerkship director’s effort goes into creating new questions for each group to be used in TBL assessments. We use OneNote very actively. However, it does not have analytical power. Thus, we are not able to effectively judge the extent of students’ involvement in our virtual learning environment.

## Conclusions

In this manuscript, we gave one example of an application of EM curriculum in a high-income developing country. We highlighted the steps that EM clerkship curriculum developers and directors should consider. We think that our reported curriculum which has details of multiple options of teaching-learning methods and assessments will guide other colleagues in developing and developed countries having immature undergraduate emergency medicine clerkship curriculum to develop or improve their own. The application of these recommendations will vary in different settings depending on resources. However, the main message is that it is important to create the curriculum content having learning outcomes relevant to the local context, and then align the TL activities, assessments, and evaluations to be in harmony. The importance and necessity of EM training in medical schools is recognized, and thus, EM training is a critical component of medical education programs [2]. Therefore, EM clerkship curricula should not only include listed topics and learning outcomes, but also all required components of a modern curriculum including TL methods, assessments, and evaluation process.

## Additional file

Additional file 1: Appendix 1. Main learning needs assessment results and proposed updates in the curriculum. Appendix 2. The undergraduate emergency medicine curriculum content, application, and sequence. Appendix 3. Assessments and grading. Appendix 4. A sample of case discussions in the OneNote platform. (DOCX 1864 kb)

## Abbreviations

CPR: Cardio-pulmonary resuscitation
EM: Emergency medicine
ILO: Intended learning outcome
i-RAT: Individual readiness assurance test
TBL: Team-based learning
TL: Teaching and learning
t-RAT: Team readiness assurance test
UAEU: United Arab Emirates University
