# Supplementary material for: Emergency medicine clerkship curriculum in a high-income developing country: methods for development and application
Source: Int J Emerg Med. 2018 Jun 7;11:31. doi: 10.1186/s12245-018-0190-y (PMC5991107; doi:10.1186/s12245-018-0190-y)
Supplement: Supplementary file 1 — Appendix 1. Main learning needs assessment results and proposed updates in the curriculum. Appendix 2. The undergraduate emergency medicine curriculum content, application, and sequence. Appendix 3. Assessments and grading. Appendix 4. A sample of case discussions in the OneNote platform. (DOCX 1864 kb) [file 12245_2018_190_MOESM1_ESM.docx]

**Additional file 1**

| **Appendix 1 - Main Learning Needs Assessment Results and Proposed Updates in the Curriculum** | | |
| --- | --- | --- |
| **Category** | **Results** | **Updates** |
| **Curriculum Content** | 1. Need for a revision of topic (chief complaint/presentation and procedure) list | Topic list revised and new curriculum topic list produced |
| **Teaching and Learning Environment** | 1. Need for additional emergency department to increase clinical exposure to different cases and procedures 2. Need for a method which can provide continuous and constructive feedback | New hospital ED which sees over 110.000 patient annually was added into clinical shift rotations  OneNote Class Notebook was chosen as online discussion board.  Electronic logbook implemented for patient and procedure encounters  Electronic supervisor clinical shift evaluation form and student shift evaluation forms created on the same platform with an electronic logbook  Gagne’s Nine Level Instruction were chosen for procedure traning |
| **Teaching Methods** | 1. Need for increased number of case discussion sessions | Teaching-day case discussion sessions increased |
|  | 1. Need for more interactive teaching sessions by adding new pedagogies | Classic teaching/lecturing was removed and flipped classroom, team-based learning along with case discussions added |
|  | 1. Need for a method which can facilitate knowledge retention | OneNote Class Notebook was chosen as online discussion board. Repeated case discussions with minor modifications were planned in this environment. |
|  | 1. Need for the implementation of smart learning / mobile technologies in and outside the classroom. | Bring your device system implemented  Socrative, google documents were used for in class exams and surveys  DartSim and Resuscitation! applications were implemented into classroom activities  OneNote Class Notebook was chosen as online discussion board. |
| **Assessments** | 1. Need for revisiting the questions for language, and their alignment with intended learning outcomes and indicative content | Weekly questions in TBL sessions, final MCQ exam questions revised under the guidance of ILOs and indicative contents.  Similar exercise was done for OSCE stations |
|  | 1. Need for adding new formative and summative assessment for students | Weekly case discussions were added into OneNote for formative assessment and feedback  Mini-CEX added into the work environment for formative assessment and feedback purposes  8 stations OSCE implemented for summative assessment |

| **Appendix 2 - The Undergraduate Emergency Medicine Curriculum Content, Application and Sequence** | | | | | |
| --- | --- | --- | --- | --- | --- |
| **Time** | **Explanation** | | | | |
| - 7 days | The undergraduate emergency medicine curriculum document and student handbook are given to all students one week before the first day of the clerkship. These documents provide curriculum content, learning outcomes, teaching and learning and assessment methods, resources for all contents as well as curriculum evaluation processes with all details. | | | | |
| - 3 days | The students take the **entry self assessment survey** regarding their confidence about ILOs three days before the course. | | | | |
| - 1 day | Students form their subgroups* and OneNote invitation is sent to students | | | | |
| **Week 1** | **Topic** | **Time given** | **Explanation** | **Application** | **Equipment, application or technology** |
| Teaching Day 1 | Review of the entry self assessment survey’s general results (5-10 minutes). The results are published in OneNote. | | | | |
| 1^st^ day of the week | Welcome and orientation | 60 minutes | Course curriculum details and its application are explained.  OneNote, e-Portfolio and Socrative application are explained. | Interactive lecture session, open to questions | - STE** |
|  | Review of the entry self assessment survey’s results regarding chest pain and related procedures (1-3 minutes) | | | | |
|  | Approach to chest pain and its specific disease entities | 75 minutes | 15 minutes - iRAT  15 minutes - tRAT  45 minutes - application exercise | TBL activity to detect learning gaps and provide opportunities to close the gaps | - Socrative for iRAT and tRAT - STE, DartSim and Resuscitation for application exercise (case discussions) |
|  | Review of the entry self assessment survey’s results regarding cardiac arrest and arrhythmia management (1-3 minutes) | | | | |
|  | Cardiac Arrest and arrhythmia management | 75 minutes | 15 minutes - iRAT  15 minutes - tRAT  45 minutes - application exercise | TBL activity to detect learning gaps and provide opportunities to close the gaps | - Socrative for iRAT and tRAT - STE and DartSim for application exercise (case discussions) |
| Teaching Day 2 | Review of the entry self assessment survey’s results regarding airway and suturing procedures (1-3 minutes) | | | | |
| 5^th^ day of the week | Airway Skills Practice | 100 minutes | Students do airway skill practices under the supervision of instructors | Skills practice with 10 minutes introduction to equipment and procedures | - Airway manikins and equipment (oxygen mask, nasal cannula, bag-valve-mask, LMA, oral and nasal airway, endotracheal tube, laryngoscopy etc.) |
|  | Suturing Skills Practice | 100 minutes | Students do suture skill practices under the supervision of instructors | Skills practice with 10 minutes introduction to equipment and procedures | - Suturing pads and equipment - Tablet and a TV/monitor to show some web based videos |
|  | Weekly group feedback session | 30 minutes | The clerkship director defines the potentially unmet learning outcomes of the week.  The clerkship director receives feedback from students regarding teaching days, clinical shifts etc.  The clerkship director provides feedback to students. | Free discussion session | - None |
|  | CLD publishes additional weekly cases and students' subgroup tasks in the OneNote. CLD reviews the student activities in OneNote reviewed and provides feedback.  Students do three clinical shifts during the 1^st^ week in non-teaching days | | | | |
| **Week 2** | **Topic** | **Time given** | **Explanation** | **Application** | **Equipment, application or technology** |
| Teaching Day 3 | Review of the entry self assessment survey’s results regarding shock and related procedures (1-3 minutes) | | | | |
| 1^st^ day of the week |  |  |  |  |  |
|  | Approach to shock and its specific disease entities | 75 minutes | 15 minutes - iRAT  15 minutes - tRAT  45 minutes - application exercise | TBL activity to detect learning gaps and provide opportunities to close the gaps. | - Socrative for iRAT and tRAT - STE, DartSim and Resuscitation for application exercise (case discussions) |
|  |  | | | | |
|  | RUSH Protocol | 60 minutes | Students are exposed to a common ultrasound protocol for patients with shock and hypotension | Interactive lecture session, open to questions | - STE |
|  | Review of the entry self assessment survey’s results regarding abdominal pain and related procedures (1-3 minutes) | | | | |
|  | Approach to abdominal pain and its specific disease entities | 75 minutes | 15 minutes - iRAT  15 minutes - tRAT  45 minutes - application exercise | TBL activity to detect learning gaps and provide opportunities to close the gaps. | - Socrative for iRAT and tRAT - STE, DartSim and Resuscitation for application exercise (case discussions) |
|  |  | | | | |
|  | Review of the entry self assessment survey’s results regarding trauma management and related procedures (1-3 minutes) | | | | |
|  | Approach to multiple trauma | 60 minutes | Students are exposed to primary and secondary survey of trauma management | Interactive discussion session, open to questions | - STE - Related online trauma management videos |
|  | E-FAST Protocol | 60 minutes | Students are exposed to a commonl ultrasound protocol in multiple trauma patients | Interactive lecture session, open to questions | - STE |
|  | Student Case Discussions | 60 minutes | Students present 10-minutes case presentations to the class. | Interactive discussion session, open to questions | - STE |
| Teaching Day 4  2^nd^ day of the week | E-FAST protocol skills practice | 100 minutes | Students do E-FAST skill practices under the supervision of instructors | Skills practice with 10 minutes introduction to equipment and procedures | - 2 ultrasound machines with human models |
|  | RUSH protocol skills practice | 100 minutes | Students do RUSH skill practices under the supervision of instructors | Skills practice with 10 minutes introduction to equipment and procedures | - 2 ultrasound machines with human models |
| Teaching Day 5  5^th^ day of the week | Individual Feedback Session | 20 minutes for each student | The clerkship director defines the potentially unmet learning outcomes of two weeks.  The clerkship director receives feedback from students regarding teaching days, clinical shifts, TBL activities, etc.  The clerkship director provides feedback to students particularly their clinical activities, ePortfolio records, TBL iRAT results, and recommendations to close the learning gaps. | Free discussion session | - None |
|  | The clerkship director publishes additional weekly cases and students' subgroup tasks in the OneNote. The clerkship director reviews the student activities in OneNote reviewed and provides feedback.  Students do three clinical shifts during the 2^nd^ week in non-teaching days | | | | |
| **Week 3** | **Topic** | **Time Given** | **Explanation** | **Application** | **Equipment, Application or Technology** |
| Teaching Day 6 | Review of the entry self assessment survey’s results regarding gastrointestinal bleeding and related procedures (1-3 minutes) | | | | |
| 1^st^ day of the week | Approach to gastrointestinal bleeding | 75 minutes | 15 minutes - iRAT  15 minutes - tRAT  45 minutes - application exercise | TBL activity to detect learning gaps and provide opportunities to close the gaps | - Socrative for iRAT and tRAT - STE, DartSim and Resuscitation for application exercise (case discussions) |
|  | Review of the entry self assessment survey’s results regarding headache and related procedures (1-3 minutes) | | | | |
|  | Approach to headache | 60 minutes | Case discussions | Interactive discussion session, open to questions | - STE - STE, DartSim and Resuscitation for application exercise (case discussions) |
|  | Review of the entry self assessment survey’s results regarding poisoned patients and related procedures (1-3 minutes) | | | | |
|  | Approach to poisoned patients | 75 minutes | 15 minutes - iRAT  15 minutes - tRAT  45 minutes - application exercise | TBL activity to detect learning gaps and provide opportunities to close the gaps | - Socrative for iRAT and tRAT - STE, DartSim and Resuscitation for application exercise (case discussions) |
|  | Review of the entry self assessment survey’s results regarding altered mental status and related procedures (1-3 minutes) | | | | |
|  | Approach to altered mental status | 60 minutes | Case discussions | Interactive discussion session, open to questions | - STE - STE, DartSim and Resuscitation for application exercise (case discussions) |
|  | Student case discussions | 60 minutes | Students present 10 minutes case presentations to the class. | Interactive discussion session, open to questions | - STE |
| Teaching Day 7  5^th^ day of the week | Cardiac arrest / arrhythmia management skills practice | 90 minutes | Students do Cardiac Arrest / Arrhythmia Management Skills practices under the supervision of instructors | Skills practice | - High fidelity manikin with monitors and related medical equipment |
|  | EFAST and RUSH protocol skills practice | 90 minutes | Students do EFAST and RUSH skill practices under the supervision of instructors | Free skills practice | - 2 ultrasound machines with human models |
|  | Airway and suturing skills practice | 90 minutes | Students do Airway and Suturing skill practices under the supervision of instructors | Free skills practice | - Airway and Suturing equipment |
|  | Weekly group feedback session | 30 minutes | The clerkship director defines the potentially unmet learning outcomes of the week.  The clerkship director receives feedback from students regarding teaching days, clinical shifts etc.  The clerkship director provides feedback to students. | Free discussion session | - None |
|  | The clerkship director publishes additional weekly cases and students' subgroup tasks in the OneNote. The clerkship director reviews the student activities in OneNote reviewed and provides feedback.  Students do three clinical shifts during the 3^rd^ week in non-teaching days | | | | |
| **Week 4** | **Topic** | **Time given** | **Explanation** | **Application** | **Equipment, application or technology** |
| Teaching Day 8 | Review of the entry self assessment survey’s results regarding respiratory distress and related procedures (1-3 minutes) | | | | |
| 1^st^ day of the week | Approach to respiratory distress | 75 minutes | 15 minutes - iRAT  15 minutes - tRAT  45 minutes - application exercise | TBL activity to detect learning gaps and provide opportunities to close the gaps | - Socrative application for iRAT and tRAT - STE, DartSim and Resuscitation applications for application exercise (case discussions) |
|  | Student case discussions | 60 minutes | Students present 10 minutes case presentations to the class. | Interactive discussion session, open to questions | - STE |
|  | Approach to fever in a child | 75 minutes | 15 minutes - iRAT  15 minutes - tRAT  45 minutes - application exercise | TBL activity to detect learning gaps and provide opportunities to close the gaps | - Socrative for iRAT and tRAT - STE, DartSim and Resuscitation for application exercise (case discussions) |
|  | Intraosseous line skills practice | 30 minutes | Students do Intraosseous line procedure under the supervision of instructors | Skills practice with 10 minutes introduction to equipment and procedures | - 2 intraosseous equipment (drill and needles) with manikins. |
|  | Weekly group feedback session | 30 minutes | The clerkship director defines the potentially unmet learning outcomes of the week.  The clerkship director receives feedback from students regarding teaching days, clinical shifts etc.  The clerkship director provides feedback to students | Free discussion session | - None |
|  | The students take the **exit self-assessment survey** regarding their post-course confidence about ILOs. The clerkship director reviews the survey results. ILOs which received less than 80% perceived achievement are added to the revision day topics.  The clerkship director publishes the exit self-assessment survey results and revision day topics in the OneNote. Student are allowed to add any content they feel they need to discuss or review  Students do one clinical shifts during the 4^th^ week in non-teaching days | | | | |
| Teaching Day 9  2^nd^ day of the week | Revision topics | 120 minutes | Revision topics are discussed one by one with the students under the guidance of learning outcomes. | Free discussion session | - STE - If needed, High Fidelity Manikin with monitors and related medical equipment, airway and suturing, intraosseous kits, ultrasound and models. |
|  | Guidance for exams | 30 minutes | Students are informed about Final MCQ exam and OSCE. | Free informative and discussion session | - None. |
|  | Skills practice | 120 minutes | Students are free to practice on procedures | Free practice session | - High fidelity manikin with monitors and related medical equipment, airway and suturing, intraosseous kits, ultrasound and models. |
| Exam Day 1  4^th^ day of the week | MCQ Exam | 150 minutes | Students receive 100 MCQs exam blueprinted according to ILOs. | 100 MCQs Exam | - Computer and Assessment Laboratory is used for the final exam. - The exam system works in intranet of the college. |
| Exam Day 2  5^th^ day of the week | OSCE | 160-200 minutes depending on the students number | Students take 8-station OSCE blueprinted according to ILOs. | 8-station OSCE. 7 minutes for each station with 2 minutes between stations | - Simulation and Skills Laboratory |
|  | Final feedback | 30 minutes | Students receive final feedback from instructors, examiners, CLD regarding their 4 week clinical performances and exam performances. This session highlights the final teaching points. | Free informative and discussion session | - None |
|  | Clerkship Survey | 15 minutes | Students take online clerkship survey regarding content, TL activities, application of the curriculum, learning environment, assessments, etc. | Anonymous survey | - Google forms |
|  |  | | | | |

*There are three balanced subgroups. Each has 4-6 students. STE = Standard Teaching Equipment (A computer, a presentation program, and a projector). TBL: Team Based Learning. iRAT: Individual Readiness Assurance Test, tRAT: Team Readiness Assurance Test. OSCE: Objective Structured Clinical Exam. MCQ: Multiple Choice Question. ILO: Intended Learning Outcomes.

| **Appendix 3 – Assessments and grading** | | |
| --- | --- | --- |
| **Assessment** | **Percent in total grade** | **Description** |
| Case presentations | 5 | Student choose one case from their encounters to present to the class. The presentation is limited to 10 minutes. Although students are advised to consider the ILOs, they are free in their choice of case. Students may choose case topics out of the curriculum content. The only requirement is to provide a teaching point relevant to emergency medicine. Students’ presentations are evaluated by CLD/tutors and peers with 20 item evaluation sheet via Google form. |
| Evaluation by supervisors | 15 | This category is divided into 2 sub-categories.  *Clinical supervisors’ evaluations*: Students need to receive 10 clinical shift evaluations from their clinical supervisors. This sub-category is responsible for 10% of total grade. Students are evaluated for punctuality, professional appearance, enthusiasm during the shift, history taking skills, physical examination skills, case reporting skills, communication skills with patients and relatives, charting skills to hospital information system. Each category is evaluated by a 5-point Likert scale from poor to excellent.  *The clerkship director /tutor evaluations*: Students are evaluated by the clerkship director /tutors for punctuality and attendance to teaching activities, active involvement in classroom and practical sessions, activity in OneNote case sharing and discussions. This sub-category is responsible for 5% of total grade. |
| Final MCQ | 20 | Students take a final exam consisting of 100 MCQs (one correct answer out of 4 options) at the end of the clerkship. The questions are blueprinted according to topics and ILOs. They are categorized into the steps of Bloom’s Taxonomy. The exam takes place at the Computer and Assessment Center of The College. |
| Final OSCE | 20 | Students take 8-station OSCE at the last day of the clerkship. 6 stations include cases applied on simulated patients or high fidelity manikin, directly observed by an examiner. Two stations contains short answer case management questions including case vignette, clinical and imaging pictures. All of the stations mainly evaluate critical actions, decision making and management processes, or skills. There are items in some stations’ sheets that evaluate professionalism. The exam takes place in the Simulation and Skills Center of the College. Examiners use e-OSCE application. |
| TBL - weekly assessments | 20 | Students take weekly exams consisting 10-20 MCQs during every TBL session via Socrative. Questions regarding chief complaint, the specific disease entities, and related procedures aligned with ILOs. The score consist of 60% of students’ iRAT score and 40% of tRAT score. The final grade from the TBL sessions is the average of all 8 TBL sessions. Students take iRAT and tRAT exams on their own mobile devices or laptop computers. |
| Patient / procedure encounter points | 20 | Students need to enrich their ePortfolio by uploading the cases and procedures they participated in. They are expected to reach a minimum of 50 cases (10 points) and 33 procedures (10 points). Students’ involvement is categorized into three levels: 1) Observation alone (observation with minimal activity), 2) Partial involvement (first assistant up to 50% activity) and 3) full involvement (start to finish care, more than 50% activity). The students self-assess and record their own level of involvement but clinical supervisors may accept, modify or cancel these records. |

TBL: Team Based Learning. iRAT: individual readiness assurance test, tRAT: team readiness assurance test. OSCE: Objective Structured Clinical Exam. MCQ: Multiple Choice Question. ILO: Intended Learning Outcomes.

| **Appendix 4: A sample of case discussions in the OneNote platform** |
| --- |
| 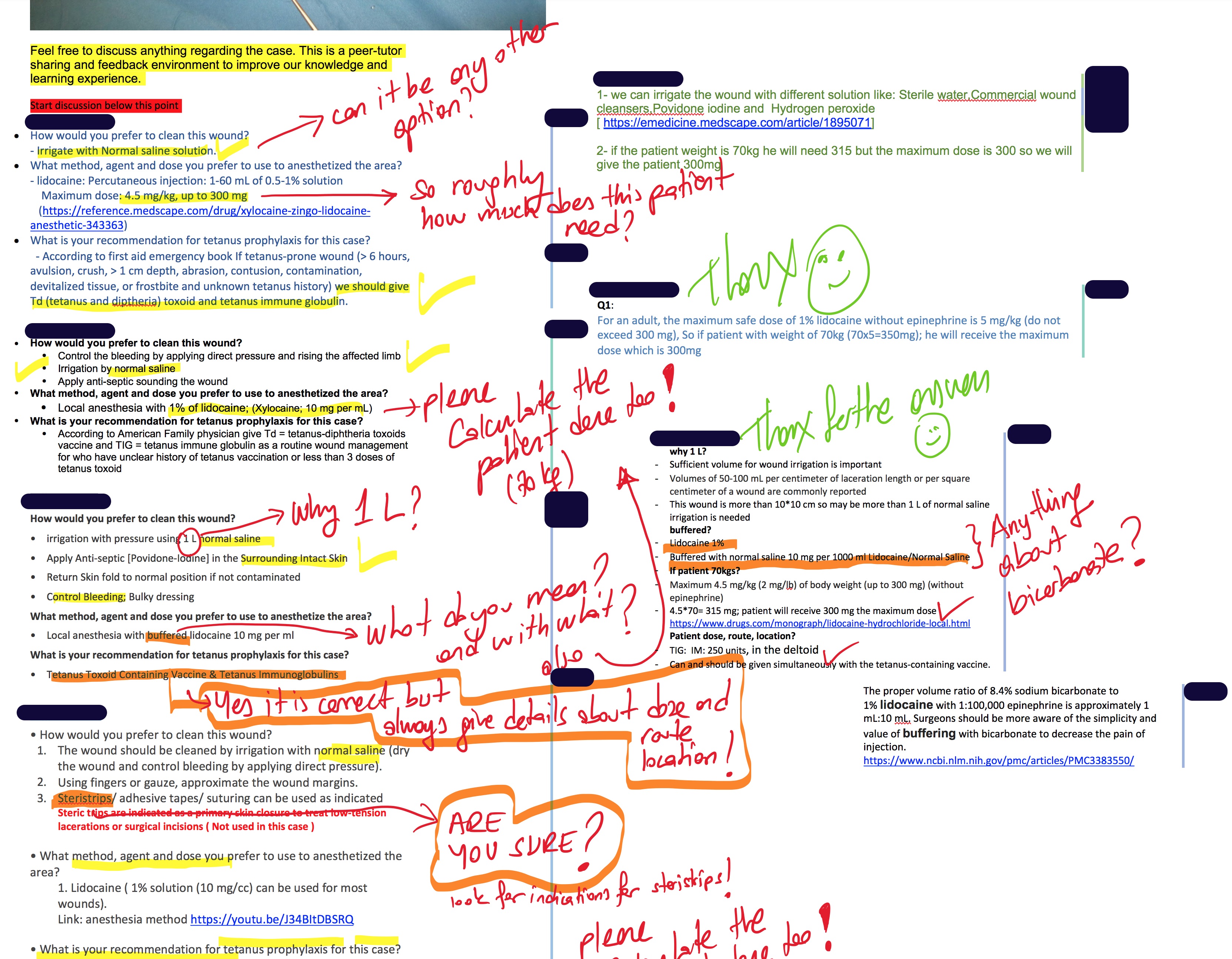 |

OneNote Class Notebook allows the students post in different formats such as handwriting, typing, audio recording. It also allows embedding videos, placing pictures, emojis, stickers. Students can work individually or as a group while they are physically separated. It also creates individual folders which can be seen by students who owns it and by their tutors. Therefore, feedback can be arranged and distributed individually if needed. Group collaboration space allows creating discussion boards which are the aim of our use. In this environment, students post their answers to cases, and these answer can be seen by all students. Tutor and peer feedbacks are also placed in this environment. Because there is no rank for these posts. Feedback can be placed just near to the original posts, and every student can easily understand where this feedback belongs to. The application does not need continuous Wi-Fi connection. The posts written in the pages can be uploaded into the system when Wi-Fi connection established.
